# An electronic alert system increases screening for hepatitis B and C and improves management of patients with haematological disorders

**DOI:** 10.1038/s41598-020-59476-4

**Published:** 2020-02-20

**Authors:** Mar Riveiro-Barciela, Paula Gubern, Luisa Roade, Pau Abrisqueta, María José Carreras, Anna Farriols, Francesc Bosch, Rafael Esteban, María Buti

**Affiliations:** 1 Liver Unit, Internal Medicine Department, Vall d’Hebron Hospital, Barcelona, Spain; 2 Centro de Investigación Biomédica en Red de Enfermedades Hepáticas y Digestivas (CIBERehd), Barcelona, Spain; 3 Department of Hematology, Vall d’Hebron Hospital, Barcelona, Spain; 4 Pharmacy Department, Vall d’Hebron Hospital, Barcelona, Spain

**Keywords:** Hepatitis B, Hepatitis C

## Abstract

Treatment of haematological disorders in patients with chronic hepatitis B or resolved infection (anti-HBc-positive) is associated with a risk of hepatitis B reactivation. Moreover, patients with chronic hepatitis C have a higher risk of haematological malignancies than general population. An electronic alert system was developed to promote screening of hepatitis B (HBV) and C (HCV) in patients starting haematological therapies. The system included screening and linkage to care and a request for testing in those without data. From March, 2017 to March, 2018 data from 420 consecutive patients with haematological diseases were included. At first prescription before the alerts, the HCV and HBV screening rate was 60.5%. Following the alerts, an additional 115 were screened, increasing the overall screening rate to 87.9%. Anti-HBc alone was detected in 57, anti-HCV in 13, and HBsAg in 2 patients. Overall, 68% of patients with any viral hepatitis markers were previously not know, and the impact was particularly important for anti-HBc detection (47/57 unknown). Nucleoside analogues were prescribed in 28 (49.1%) anti-HBc-positive and the 2 HBsAg-positive patients. Prospective follow-up with HBV DNA and HBsAg testing showed no cases of HBV reactivation. An estimated 1.2 HBV reactivations were avoided as consequence of the alert system. In summary, an electronic alert system increased viral hepatitis screening in patients receiving haematological treatment and led to improvements in the management of these patients, including avoided HBV reactivation.

## Introduction

Viral infection by hepatitis B and C plays an important role in patients with haematological malignancies. Individuals with chronic hepatitis B virus (HBV) infection, (i.e., HBsAg-positive), and even those with resolved infection (HBsAg-negative/anti-HBc-positive) are at risk of HBV reactivation when receiving chemotherapy^1^, especially those treated with rituximab-containing regimens^2^. The importance of HBV screening lies in the potential severity of HBV reactivation, ranging from asymptomatic HBV DNA elevation to the development of acute liver failure with an extremely high mortality rate^3^, which results in a poorer prognosis^4^. To prevent reactivation, it is important to determine the HBV status prior to immunosuppressive therapy to identify individuals who will benefit from HBV prophylaxis^5^. However, the rate of testing for viral hepatitis in the haematological population is relatively low. The reported HBV screening rate in patients with haematological malignancies in a large study in a US cancer centre was as low as 66% over a 7-year period^6^. In this line, data from Spain have shown the beneficial impact of an electronic alert system for physicians prescribing biological drugs, to remind them of the possibility of HBV reactivation. In one study, the HBsAg screening rate rose from 47% to 94% and anti-HBc screening from 30% to 85%^7^.

As to hepatitis C virus (HCV) infection, there is a well-established association between this condition and the development of lymphoma, with a reported risk 60% higher in HCV-infected individuals than in uninfected controls^8^. Diffuse large B-cell lymphoma (DLBCL), lymphoplasmacytic lymphoma and marginal zone lymphoma are the most commonly linked malignancies^9^.

In view of the high prevalence of HCV in haematological patients and the potential risk of HBV reactivation in this population, we developed an electronic alert system (EAS) to increase the screening rate in patients with haematological malignancies undergoing specific therapy. This application is linked to the hospital prescription software and requires introduction of the patients’ HBV (HBsAg and antiHBc) and HCV serology results every time a new therapy scheme is prescribed. The data are transferred by e-mail to the Liver Department, which facilitates referral of individuals with positive serology and requests testing in patients without this information. The aim of this study was to analyse the impact of the EAS in the first year of its implementation on the rate of HBV and HCV screening in patients with haematological malignancies undergoing specific therapies. In addition, the estimated number of HBV reactivations avoided as a result of the EAS was calculated.

## Results

### Patients’ baseline characteristics

The study included 420 consecutive patients with haematological diseases. Baseline characteristics are summarized in Table 1. Mean age was 63 years, 233 patients were men, and most were Caucasian. The most common malignancies were DLBCL, multiple myeloma and follicular lymphoma. Four (1%) patients were anti-HIV-positive and 18 (4.3%) reported previously known viral hepatitis.

**Table 1:** Baseline characteristics of patients undergoing chemotherapy for hematological malignancies.

**Male gender**, n(%)	233 (55.5%)
**Race**, n(%)	
Caucasian	398 (94.8%)
Asian	10 (2.4%)
Hispanic	8 (1.9%)
African	4 (1%)
**Age**, mean ± SD	63 ± 16
**Most common hematological malignancy**, n(%)	
Diffuse large B cell lymphoma	72 (17.1%)
Multiple mieloma	65 (15.5%)
Follicular lymphoma	57 (13.6%)
Acute leukemia	41 (9.8%)
Chronic myeloid leukemia	37 (8.8%)
Hodgkin lymphoma	22 (5.2%)
Myelodysplastic syndrome	22 (5.2%)
Chronic lymphocytic leukemia	18 (4.3%)
**Anti-CD20-containing regimens**, n(%)	166 (39.5%)
**Tyrosine-kinase inhibitors**, n(%)	64 (15.2%)
**HIV infection**, n(%)	4 (1%)
**Previously known viral hepatitis**, n(%)	18 (4.3%)*
Anti-HCV positive	10
HBsAg positive	1
Anti-HBc positive	11

*4 patients were both anti-HCV and anti-HBc positive.

There were no missing data for any of the variables.

### Performance of the electronic alert system

#### Impact on viral hepatitis screening

At the first prescription, the serological panel was completed in 254 (60.5%) patients. The screening rate clearly differed according to the type of treatment. The highest rates of completed panels were in patients receiving anti-CD20-containing regimens (69% vs 55%, p = 0.002) or therapy for multiple myeloma (69% vs 59%, p = 0.075); a very low rate was seen in relation to tyrosine-kinase inhibitors (29.7% vs 66%, p < 0.001). The haematological malignancy also had an impact on screening (p < 0.001), with the highest percentage of completed panels seen in patients with primary cerebral lymphoma, DLBCL, and Burkitt’s lymphoma (100%, 83.3%, and 80%, respectively) and the lowest in those with chronic myeloid leukaemia (CML) (16.2%).

**Figure 1:**
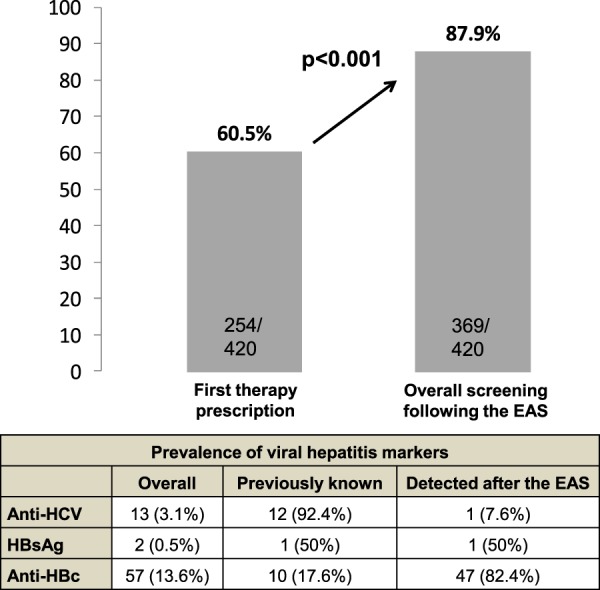
Impact of the electronic alert system (EAS) on screening for viral hepatitis markers. At the start of new therapies for haematological patients, viral hepatitis markers were tested in 60.5% of the total. Following the alerts provided by the EAS, the screening rate significantly increased to 87.9%.

Following the alert, 115 additional cases were tested for viral hepatitis, increasing the overall screening rate to 87.9% (Fig. 1). Percentage of completed panel of screening according to the epidemiological, clinical, and therapeutic factors of patients is summarized in Table 2. Overall, anti-CD20-containing regimens and DLBCL patients were the ones with the highest screening rates, whereas those receiving tyrosine-kinase inhibitors or and patients CML had the lowest. Mean age of patients with complete viral panel did not differed to those without (62.8 vs 63.5 years, p = 0.7).

**Table 2:** Overall viral hepatitis screening rate according to epidemiological, clinical and therapeutic factors.

Factor	N Patients	Screening rate	p value*
Male gender	233	90.1%	0.075
Age ≥65 years	227	88.5%	0.374
Caucasian race	398	87.9%	0.514
DLBCL	72	97.2%	0.003
Multiple myeloma	65	93.8%	0.074
Chronic myeloid leukemia	37	48.6%	<0.001
Anti-CD20-containing regimens	166	93.4%	0.003
Tyrosine-kinase inhibitors	64	62.5%	<0.001

*Comparison of rate of screening in contrast with the rest of the cohort.

#### Prevalence of viral hepatitis markers

Anti-HBc alone was detected in 57 patients, anti-HCV in 13, and HBsAg in 2 patients. Among these patients, 47 with anti-HBc alone, 1 with HBsAg, and 1 with anti-HCV were newly detected by the EAS (Fig. 1). Therefore, the status of 68% of patients testing positive for viral hepatitis markers had been unknown previously.

#### Management of patients testing positive for HBV markers

**Figure 2:**
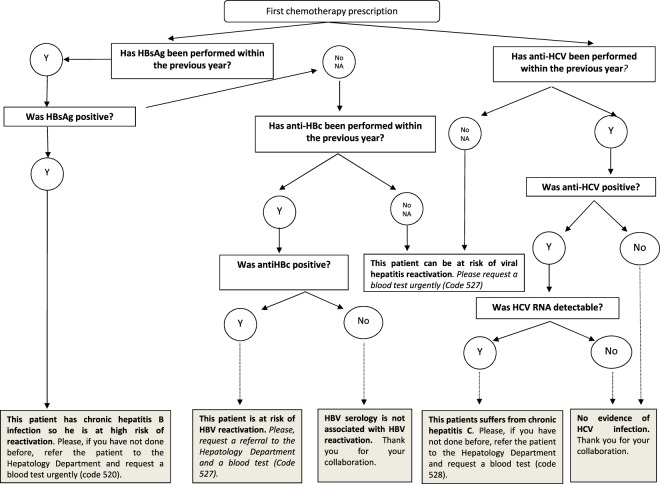
Flow chart showing the electronic alert system (EAS) for screening of viral hepatitis markers in patients undergoing haematological therapy. In collaboration with the Haematology and Pharmacy Departments, a software linked to the prescription application was created to remind physicians and facilitate testing for HBsAg, anti-HBc, and anti-HCV in haematological patients. In patients with positive results or an absence of data, the EAS automatically sends an e-mail to the Hepatology Department. To facilitate the physicians’ work and obtain relevant data, 3 preconfigured test requests were created, one for patients with chronic hepatitis B, one for those with chronic hepatitis C, and one for anti-HBc-positive patients and those lacking data on viral hepatitis markers. NA, not available.

**Figure 3:**
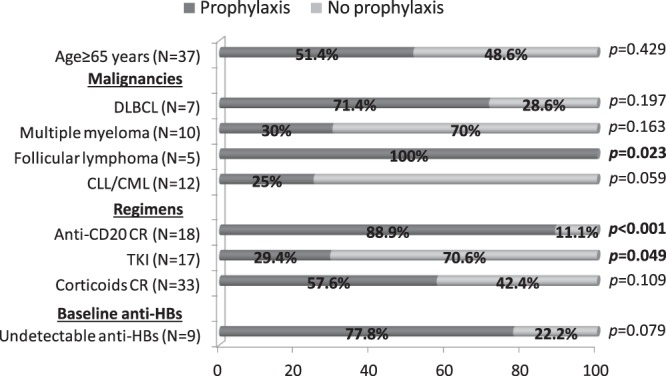
Use of antiviral prophylaxis for anti-HBc-positive patients receiving treatments for haematological conditions. Prescription of antiviral prophylaxis to avoid HBV reactivation differed according to the underlying malignancy and therapy scheme. Screening was clearly higher in patients treated with anti-CD20-containing regimens and those with follicular lymphoma, and was lower in TKI-treated patients. DLBCL, Diffuse B cell lymphoma; CLL/CML, chronic lymphocytic/myeloid leukaemia; CR, containing regimen; TKI, tyrosine-kinase inhibitors.

Despite the EAS recommendations (Fig. 2), HBV DNA analysis was performed in only 43 (75.4%) of the 57 anti-HBc-positive individuals, and it was undetectable in all cases. Antiviral prophylaxis was prescribed in 28 (49.1%) patients. Patients receiving anti-CD20-containing regimens and those with the malignancies most commonly treated with these drugs (DLBCL and follicular lymphoma) were statistically more prone to receive antiviral prophylaxis (Fig. 3). There was also a trend to prescribe prophylaxis in patients with low baseline levels of anti-HBs (<10 mIU/mL), although mean baseline anti-HBs titres did not differ between those who received antiviral prophylaxis or not (274.4 vs 337 mIU/mL, p = 0.556), neither did age (67.3 vs 69.2 years, p = 0.5). Patients with CLL or CML and those treated with tyrosine-kinase inhibitors tended not to receive antiviral prophylaxis. Among patients receiving tyrosine-kinase inhibitors, the underlying haematological disease had an impact on the prescription rate of antiviral prophylaxis, with only 2/40 (5%) patients with CLL or CML receiving prophylaxis versus 4/11 (26.7%) with the remaining indications (p = 0.041), in particular patients who had undergone stem cell transplantation.

Concerning the two HBsAg-positive patients, the one diagnosed by the EAS had a detectable viral load (154 IU/mL), and antiviral prophylaxis with nucleos(t)ide analogues (NUCs) was prescribed. The other patient had HBeAg-negative chronic hepatitis and was already receiving a NUC, which was maintained. From the 57 anti-HBc positive patients, 47 were diagnosed after the EAS and 25 (53.2%) received prophylaxis with NUCs.

#### HBV reactivation within the first year of the EAS

Ten out of 57 antiHBc-positive patients died during follow-up. However, in 3 of these subjects data on HBV reactivation was available during therapy. Therefore, prospective data on HBsAg and HBV DNA was available in 50 anti-HBc positive patients. No cases of HBV reactivation were observed during a mean follow-up of 11 ± 4 months (time from the first prescription to the last analytical control). Twenty-five (50.0%) of these patients were receiving antiviral prophylaxis. There were no differences between baseline and follow-up anti-HBs titres (321 vs 292, respectively, p = 0.232). The two HBsAg-positive patients received antiviral treatment and achieved virological suppression (undetectable HBV DNA), which was maintained during follow-up.

#### Estimated number of HBV reactivations avoided by the EAS

According to previously published meta-analyses and controlled trials, the estimated risk of HBV reactivation in anti-HBc-positive patients receiving treatment for hematological malignancies is 2.4% in those with antiviral prophylaxis and 7.2% in those without^10,11^. Based on these rates, the number needed to treat (NNT)—that is, the number of antiHBc-positive patients in this setting who should receive antiviral prophylaxis to avoid 1 case of HBV reactivation—would be 20.8 (95% CI -18.2-13.9). As 25 antiHBc-positive patients diagnosed by the EAS received antiviral prophylaxis, the estimated number of avoided HBV reactivations was 1.2.

#### Management of anti-HCV-positive patients

Overall, 13 patients tested positive for anti-HCV. HCV RNA was analysed in all cases and 2 patients had a detectable viral load, one with Burkitt’s lymphoma and the other with follicular lymphoma. These two patients were later treated with direct-acting antiviral agents and achieved a sustained virological response. Seven of the 11 (63.6%) patients with undetectable HCV RNA had been treated previously. Four (36.4%) were liver-transplant recipients undergoing chemotherapy for post-transplant lymphoproliferative disease (PTLD).

## Discussion

Implementation of an electronic alert system had a positive impact on the viral hepatitis screening rate in haematological patients receiving specific therapies, with an increase from 60.5% to 87.9% of patients. The importance of the EAS lies in the diagnosis of previously unknown viral hepatitis markers, particularly HBV. In our cohort, 80% of the 57 patients with anti-HBc were diagnosed following the alert provided by the EAS. This prompted the start of antiviral prophylaxis in half the patients, which avoided an estimated 1.2 cases of HBV reactivation.

Alert systems focusing on HBV have been described previously^7,12^. In the present study, addition of HCV to the test panel in our EAS was also found to be useful for diagnosing new cases of chronic hepatitis C, which led to the start of treatment with direct-acting antiviral agents and sustained virological response. Currently, considerable effort worldwide is devoted to promoting HCV screening programs (including microelimination programs in targeted populations) to achieve elimination of the virus. Implementation of an EAS, mainly in those at high-risk such as haematological patients, could be a useful step toward eliminating HCV in hospitals.

Since emergence of the FDA warning on HBV reactivation in patients receiving anti-CD20 drugs^13^, the risk of this occurrence in haematological patients undergoing chemotherapy or targeted therapies has been extensively studied. The hazard depends on the underlying disease and therapy scheme, but mainly on HBV status^2,14^. Hence, testing for viral hepatitis markers before starting these drugs is vital to identify patients at risk of HBV reactivation, who may be candidates for antiviral prophylaxis. In this regard, the use of alert systems has been explored to improve screening rates^7,12^. Sampedro *et al*. reported that use of a computerized physician order entry-based system for patients treated with biologic agents led to a screening rate for HBV markers greater than 85%^7^, a value comparable to the overall percentage of testing in our cohort after the EAS alerts. Another important differential aspect of our EAS is its link to the Hepatology Department, which facilitated linkage to care in patients with chronic hepatitis B or C and the request for viral markers in patients without this data.

The results of this real-world study bring to light the differing perception of viral hepatitis risk depending on the patient’s therapeutic regimen and underlying malignancy. It is well-established that rituximab-containing regimens, mainly rituximab with cyclophosphamide, doxorubicin, vincristine, and prednisone (R-CHOP), are strongly linked to a high risk of HBV reactivation^2,4,15^. However, in recent years other chemotherapy schemes, such as those for multiple myeloma, have also been associated with a risk of HBV reactivation^16,17^. In our cohort, the malignancy associated with the lowest screening rate was CML. The risk of HBV reactivation associated with tyrosine kinase inhibitors has been scarcely explored, and although it is considered to be moderate^18^, the increasing use of these drugs has led to a recent rise in the reported cases of reactivation^19–23^.

This observational study was carried out in the first year of EAS implementation, with prescription of antiviral prophylaxis left to the discretion of the treating haematologist in patients testing positive only for anti-HBc. This design also provided interesting data on the awareness of physicians regarding the risk of HBV reactivation in this population. In our cohort, and in line with current international guidelines, most patients prescribed anti-CD20-containing regimens received prophylaxis, including all patients with follicular lymphoma. However, our data indicated an alarmingly low concern about reactivation risk in anti-HBc-positive patients receiving tyrosine kinase inhibitors or schemes including corticosteroids. Apart from R-CHOP and anti-CD20-containing regimens, the risk of HBV reactivation in anti-HBc-positive hematological patients is poorly stratified, especially concerning the latest approved drugs. In the case of tyrosine kinase inhibitors, although a few case reports have shown a risk of HBV reactivation, including development of acute liver failure^21,24^, only one retrospective study including 10 anti-HBc-positive patients assessed this potential hazard. No cases of HBV reactivation were found after a mean follow-up of more than 3 years, despite an absence of antiviral prophylaxis^25^. In our cohort, although the concern for screening was low in the population receiving tyrosine kinase inhibitors (62.5%), the complete testing data showed 17 anti-HBc-positive individuals. Among these, 12 (70.6%) did not receive prophylaxis and there were no cases of reactivation during the first year of therapy.

Regarding the prevalence of viral hepatitis, 3.2% of patients tested anti-HCV positive, a value 3 times higher than the 0.99% recently reported from the same area^26^. Only 2 of the anti-HCV-positive patients had a detectable viral load. These findings can be explained by the fact that several anti-HCV-positive liver transplant recipients with PTLD had already achieved sustained virological response. As was expected, the prevalence of HBsAg and anti-HBc was similar to previously reported values^26,27^.

Our study has some limitations. As antiviral prophylaxis for HBV was prescribed at the discretion of the treating haematologist, its use was not homogeneous in the cohort. However, this factor resulted in interesting data regarding the concern for HBV reactivation in real-world daily clinical practice according to the underlying haematological condition and therapy prescribed. Moreover, usefulness of this alert system could be extrapolated to other specialties that prescribe immunosuppressant drugs such as oncology, rheumatology or dermatology.

In conclusion, the use of an electronic alert system had a positive impact on the viral hepatitis screening rate in patients receiving haematological therapy. This, in turn, led to an improvement in the management of these patients, with diagnosis of previously unknown cases of chronic viral hepatitis and possible avoidance of HBV reactivations.

## Methods

### Study design

An EAS was developed to remind physicians of the need for HBV and HCV testing in certain patients. The EAS was linked to the hospital’s prescription software and requested HBsAg, anti-HBc, and anti-HCV results when specific haematological drugs were prescribed. The physician was informed about the potential risk of HBV reactivation and the need to refer the patient to a hepatologist according to the scheme summarized in Fig. 2. In patients testing HBsAg or antiHBc positive and those with unknown status, the system automatically sends an e-mail to the Hepatology Department with the patient’s coded data. Furthermore, to facilitate the physicians’ work and achieve relevant data, 3 preconfigured blood tests were created: one to complete the study of HBsAg-positive individuals (*Code 520*, which included a haemogram, biochemistry, clotting, quantitative HBsAg, HBV DNA, HBeAg, anti-HBe, anti-hepatitis D virus, human immunodeficiency virus (HIV) serology, and anti-HCV), another for anti-HBc positive or unknown serological status (*Code 527*, including HBsAg, anti-HBc, HBV DNA, and anti-HBs titers), and a third for those with detectable HCV RNA (*Code 528*, haemogram, biochemistry, clotting, HIV serology, and HCV genotype). Whenever a new treatment schedule was prescribed, the computerized system requested the patient’s viral hepatitis status if it had not been previously introduced. If data were lacking and viral hepatitis screening had not been performed within the first 3 months after the therapy prescription, the viral hepatitis panel was added to the patient’s next scheduled blood test.

Prior to implementation of the EAS, Haematology Department physicians underwent training sessions to highlight the importance of hepatitis B and C screening, the rationale to set up this system and to inform about the functioning of the EAS. During the first year of the system, this prospective, observational study was carried out to evaluate the performance of the EAS according to the following items:

- Number of patients whose viral hepatitis status was checked after the EAS warning. All patients lacking data at the first prescription were checked for later viral hepatitis screening requests from the Haematology Department.
- Number of patients with newly diagnosed viral hepatitis markers.
- Number of HBV prophylaxis therapies initiated as a result of the EAS (newly diagnosed HBsAg- or anti-HBc-positive patients) and estimation of the number of HBV reactivations avoided because of the EAS.
- Number of HBV reactivations within the first year of the EAS.

This study was approved by the Vall d’Hebron Hospital ethics committee and was conducted in compliance with the principles of the Declaration of Helsinki, Good Clinical Practice guidelines, and local regulatory requirements. Since screening of viral hepatitis markers is considered within recommended daily clinical practice, no informed consent was requested in agreement with the Hospital ethics committee.

### Definition of HBV reactivation

HBV reactivation was defined according to the AASLD guidelines as a ≥ 2 log increase in baseline HBV DNA in HBsAg-positive patients, or reappearance of HBsAg or detectable HBV DNA in patients who had been previously HBsAg-negative/anti-HBc-positive (reverse seroconversion)^28^.

### Patient selection

All haematological patients receiving specific treatment (including monoclonal antibodies in monotherapy) from 1 March, 2017, the date the EAS was launched, to 1 March, 2018, were included.

### Data collection

In all cases, epidemiological (race, date of birth, sex), clinical (malignancy, treatment regimen, previously known viral hepatitis infection, prior treatment for viral hepatitis, antiviral therapy), and serological (HBsAg, anti-HBc, anti-HCV, and HIV if available) data were recorded, as well as antiviral treatment for chronic hepatitis C and nucleos(t)ide analogue prophylaxis for HBsAg- or anti-HBc-positive patients. These treatments were prescribed at the discretion of the haematologist, except for patients referred to the Hepatology Department and those with chronic hepatitis B, who were treated in all cases.

### Laboratory measurements

Serological markers for HBV (HBsAg, anti-HBc, and anti-HBs) and HCV (anti-HCV) were analysed by commercial enzyme immunoassays. The lower limit of quantification of anti-HBs was 10 mIU/mL and the upper limit, 500 mIU/mL. Serum HBV DNA was quantified by PCR with a COBAS 6800 HBV test (Roche Diagnostics, Mannheim, Germany): lower limit of quantification, 20 IU/mL and lower limit of detection, 10 IU/mL.

### Statistical analysis

Normally-distributed quantitative variables were compared with the Student *t* test and expressed as the mean ± standard deviation (SD). Variables with a non-normal distribution were analysed with the Mann-Whitney *U* test and expressed as the median and interquartile range. Categorical variables were compared using the chi-square or Fisher exact test, as appropriate, and expressed as frequencies and percentages. *P* values < 0.05 were considered statistically significant. The impact of the EAS was estimated using the number needed to treat (NNT), based on the number of patients who initiated antiviral prophylaxis, and the 95% confidence interval was calculated using the Newcombe method^29^. All analyses were carried out using IBM SPSS, 20 (SPSS Inc., Armonk, NY, USA).
